# Disrupted Attention to Other’s Eyes is Linked to Symptoms of ADHD in Childhood

**DOI:** 10.1007/s10578-022-01316-9

**Published:** 2022-01-17

**Authors:** Matilda A. Frick, Karin C. Brocki, Linda Halldner Henriksson, Johan Lundin Kleberg

**Affiliations:** 1 Division of Emotion Psychology, Department of Psychology, Uppsala University, Box 1225, 751 42 Uppsala, Sweden; 2 Department of Clinical Science, Child and Adolescent Psychiatry, Umeå University, Umeå, Sweden; 3 Department of Medical Epidemiology and Biostatistics, Karolinska Institutet, Stockholm, Sweden; 4 Department of Clinical Neuroscience, Centre for Psychiatry Research, Karolinska Institutet, & Stockholm Health Care Services, Region Stockholm, Stockholm, Sweden; 5 Department of Molecular Medicine and Surgery, Karolinska Institutet, Stockholm, Sweden

**Keywords:** Attention-deficit/hyperactivity disorder (ADHD), Eye tracking, Face perception, Anxiety, Internalizing, Externalizing symptoms

## Abstract

Attention-deficit/hyperactivity disorder (ADHD) is associated with impaired social interaction. Other’s eyes are important for understanding the social world. Here, we examined concurrent and longitudinal links between attention to other’s eyes and symptoms of ADHD and comorbid externalizing and internalizing symptoms. Eighty-two 8 to 13-year-old children (40% with ADHD) participated. The latency to a first gaze shift to and away from the eye region of human faces, when primed to look at either the eyes or the mouth, was recorded with eye tracking. Parents rated ADHD, externalizing and internalizing symptoms at the time of testing and at 2-year follow-up. The results show that longer looking at the eyes before reorienting was specifically associated with concurrent and future symptoms of inattention, even when accounting for comorbid symptoms. We conclude that the temporal microstructure of attention to other’s eyes is altered in children with symptoms of ADHD, which may contribute to social impairments.

## Introduction

Attention-deficit/hyperactivity disorder (ADHD) is associated with impairments in multiple life domains, including school and occupational performance, peer relations and increased risk for delinquency such as alcohol and substance abuse [1–3]. Although not part of the diagnostic criteria, social interaction impairments are common in ADHD across the life span [4]. For example, children with ADHD are often excluded from peer groups because of difficulties with turn-taking, behavioral regulation during play [5, 6] and regulation of both positive and negative affect [7]. For humans, faces are one of the most important sources of learning about the social environment [8]. Disrupted attention to faces could therefore be an underlying mechanism of the social interaction impairments observed in ADHD. A small literature has examined how children with ADHD attend to faces. For example, previous studies have reported that children with ADHD respond to faces with altered gaze behavior [9], autonomic [10] and brain activity [11, 12]. Recently, Gui and colleagues [13] reported that 14-month-old toddlers who had elevated symptoms of ADHD at a follow-up looked longer at images of faces among distractors before reorienting, suggesting difficulties with disengagement of attention. These studies do not indicate that face-perception atypicalities in ADHD depend on the emotional expression of the face [e.g. 9, 10].

The eye region is the most informative part of the face [e.g. 14]. Recent research suggests that children with ADHD may show specific impairments related to processing of other’s eye gaze. For example, children with ADHD often fail to attend to others’ eyes during emotion recognition [15] and are not using others’ gaze direction to guide their attention [16]. Consequently, intervention programs teach parents to increase periods of sustained eye contact with their children with ADHD as a means of increasing compliance [17].

Information from the eye region is believed to be a major source of learning and experience-dependent development of the social brain [14] and could therefore contribute to the developmental course of ADHD. However, to our knowledge, no longitudinal studies have so far examined visual attention to eyes as a longitudinal predictor of ADHD symptoms. Altered face perception may be a common phenotypic characteristic of ADHD and autism spectrum disorder (ASD) [18], as reduced attention to faces in the presence of competing non-social information, reduced eye contact, and a diminished ability to understand information expressed in the eye region is commonly seen in ASD [19].

Visual scanning of faces follows a relatively uniform sequence. Initially, human eyes trigger quick and reflexive gaze shifts towards the eyes, a process called social orienting [14]. Subsequently, gaze is reoriented from the eyes to other aspects of the face (typically the mouth) or to the focus of the other person’s gaze. Disruptions to both these processes (orienting and reorienting) could potentially interfere with social perception and interaction [20]. It is therefore important not only to examine whether ADHD and associated symptom dimensions are linked to atypical attention to eyes, but also to establish at what stage. That is, how early in the attentional sequence potential deviations can be observed and whether orienting towards the eyes, away from the eyes or both is affected. Further, this pattern could vary as a function of the depicted emotion in the face. For instance, theories of social attention in children with anxiety disorders have focused on three processes – *vigilance*, *avoidance*, and *delayed disengagement* [21, 22]. Vigilance would be manifested as rapid gaze shifts towards potentially threatening stimuli such as angry faces, avoidance as a tendency to orient away from the same stimuli, and delayed disengagement as increased dwell time before reorienting, perhaps reflecting problems with shifting attention from potential threats. These theories have mainly been tested in studies comparing the relative allocation of attention between whole faces with a threatening expression and various non-social control stimuli [22, 23], and not in relation to eye gaze specifically. A recent study of adolescents with social anxiety disorder [20] found that this group differed from healthy controls by taking longer time to reorient from eyes, supporting the *delayed disengagement* hypothesis. It is not known whether a similar pattern of attention pertains also to other forms of anxiety symptoms, such as generalized anxiety, when comorbid with ADHD.

ADHD is highly overlapping with externalizing symptoms of oppositional defiant disorder (ODD) and conduct disorder (CD) and internalizing symptoms such as anxiety. ODD refers to a pattern of anger, irritability and defiance towards caregivers and other adults with a typical onset in early childhood [24]. CD refers to a more severe pattern of antisocial behavior, characterized by lack of respect for others, reduced empathy and rule breaking [24]. Different anxiety disorders are usually highly correlated and not always easily distinguishable, suggesting that they are closely linked to a common internalizing factor [25]. Generalized anxiety is characterized by excessive and widespread worrying [24]. Symptoms of generalized anxiety commonly debut in childhood and are closely linked to trait neuroticism, which represents a broad risk factor for internalizing disorders [26], making it a suitable construct for the current study. These co-occurring externalizing and internalizing symptom dimensions are both associated with atypical eye gaze processing [20, 27]. It is therefore not clear whether the link between ADHD symptoms and atypical attention to others’ eyes is due to core ADHD difficulties or to comorbid externalizing and internalizing symptoms. However, scarce research of facial emotion recognition have suggested that the challenges children with ADHD face within this area may be better explained by comorbid externalizing symptoms than by ADHD symptoms per se [e.g. 15].

There is some evidence that symptoms of externalizing disorders are related to a *reduced tendency to orient* to eyes during free-viewing tasks, and that this in turn is related to impaired emotion recognition [e.g. 15]. For example, Dadds et al. [27] reported that boys with callous unemotional traits, a severe form of externalizing symptoms characterized by low emotionality, poor affective empathy, and proactive aggression, were less likely to orient to others’ eyes, which in turn affected their emotion recognition. Other studies have suggested that personality traits associated with enhanced reward sensitivity and interpersonal aggression are linked to prolonged periods of eye contact once it is established [28]. Given the relationships between externalizing symptoms, enhanced reward sensitivity, and interpersonal aggression, externalizing symptoms may be connected to delayed orienting away from eyes, although it is not clear from the previous literature whether this effect would be modulated by the emotional expression of the face.

The developmental correlates of altered eye gaze in ADHD remains largely unexamined. Human eyes are arguably one of the most important sources of social learning of the environment [14]. Consequently, maladaptive attention to other’s eyes during childhood could potentially interfere with social learning, thereby exacerbating core ADHD and comorbid symptom levels over time and as such contribute to non-optimal development. On the other hand, flexible attention to others’ eyes could potentially increase opportunities for symptom reduction through adaptive social learning. In both scenarios, attention to others’ eyes would be expected to predict subsequent change in ADHD, externalizing, and internalizing symptoms but in different directions. There is currently a lack of studies examining this matter.

Even though ADHD, ODD/CD, and generalized anxiety refer to diagnostic categories, it has been argued that empirically psychopathological constructs are best conceptualized as dimensional traits [29]. As such, obtaining a dimensional perspective adds both statistical power and a more fine-grained understanding of connections between symptoms dimensions that are interrelated yet separable by different developmental trajectories and associated risks [30, 31].

To sum up, previous studies have linked symptoms of ADHD and comorbid symptoms of ODD/CD and anxiety to atypical eye contact, but many questions remain. For instance, the specificity of these atypicalities to different symptom dimensions; the sequency stage at which they occur; the direction of the effects (enhanced vs. diminished eye contact); and the extent to which atypical eye contact predict change in symptom levels across development. We used an eye preference task (EPT) in which a group of school-aged children (aged 8–13 years), oversampled for ADHD-diagnoses (~ 40% had a previous diagnosis of ADHD) were primed to look towards the eyes or the mouth of depicted faces expressing different emotions (i.e. angry, happy, and neutral). Eye preference was operationalized as the latency to *orient away* (OA) from the eyes when the initial point of gaze was in the eyes region and the latency to *orient towards* (OT) the eyes when the initial point of gaze was in the mouth region. We set out to examine the orienting measures globally (i.e. a composite of all expressed emotions) and separately for each expressed emotion.

We examined the following research questions:

1. Is eye preference concurrently and specifically (i.e., with control for the other symptom domains) related to symptoms of ADHD, ODD/CD, and generalized anxiety?
2. Is eye preference longitudinally and specifically related to symptoms of ADHD, ODD/CD, and generalized anxiety at follow-up two years later (T2)?
3. Is eye preference related to the change in symptoms of ADHD, ODD/CD, and generalized anxiety between T1 and T2 (with and without control for the other symptom domains at T1)?

As for the direction of effects, we expected symptoms of ODD/CD to be linked to delayed orienting away from the eyes when the eye region was primed (OA) and delayed orienting towards eyes when the mouth was primed (OT). Further, we expected symptoms of generalized anxiety to be related to delayed orienting away from eyes when the eye region was primed (OA). No directed hypotheses were specified for the other research questions or for specific emotions due to inconsistencies in the previous literature.

## Method

### Participants

An initial sample of 84 children aged 8 to 13 years old participated in the study at T1. Due to technical errors with the EPT paradigm, the final sample consisted of the 82 individuals (M^age^ = 10.49 years, SD = 1.36; 59 boys, 72%) who were successfully assessed with the EPT. Thirty-four children (41%) had a diagnosis of ADHD (24 boys, 71%). At T2, which took place 2 years later, 67 children remained in the study (retention rate 82%; 46 boys, 69%; M^age^ = 12.57 years, SD = 1.42), of which 27 (40%) had a diagnosis of ADHD (18 boys, 67%). The remaining children had slightly higher socio-economic status (SES) compared to the dropouts (4.23 vs. 3.52, t(79) = 2.46, *p* = 0.016), but did not differ on any of the other study variables (*p*s > 0.16). The children with ADHD were recruited through ads in the local newspaper (n = 10), on Facebook (n = 2), through interest groups (n = 4), and through child-psychiatry and pediatric outpatient clinics (n = 18). Diagnostic status was confirmed through medical records in 15 cases, for the remaining 19 cases the parents reported that their children had been formally assessed in regular child psychiatric or pediatric care and had received an ADHD diagnosis. The children without a diagnosis were recruited through the population registry and information letters about the study were sent to 1,000 families in the local area. Of 116 interested responders, 50 children without psychiatric disorders, matched with the ADHD group for age, sex, and SES were enrolled in the study.

Of the children with ADHD, 80% were on regular medication for ADHD (methylphenidate, atomoxetine, dexamphetamine, or extended-release guanfacine). Individuals with stimulant medication were asked to refrain from medication on the day of assessment, yet 10 (29%) were medicated during the EPT. These visits were still completed as the test leader or the parent did not deem it feasible to postpone them until after a washout phase. Analyses showed that medication on the day of testing was unrelated to all EPT measures and was therefore left out of further analyses. Four individuals reported a comorbid disorder of autism spectrum disorder. Removing these did not change the results in a profound way and they were therefore kept in the final sample. As for other comorbid disorders, three participants reported an anxiety disorder, one a sleep disorder, and one a language disability. The regional ethics board (EPN 2014/285, Uppsala, Sweden) approved the study. All legal guardians gave written informed consent, and all children gave oral informed consent to participation.

### Procedure

The study consisted of a 2-h visit to the Department of Psychology, Uppsala University or to Kista BUMM, a pediatric outpatient clinic in Kista, Stockholm. The children were assessed with the EPT and various other tasks presented elsewhere [10, 32, 33]. Parents (mothers [n = 62, 75.6%], fathers [n = 2, 2.4%], or both parents together [n = 19, 23.2%]) completed questionnaire data on symptom levels at T1 and T2. For participation, the families received gift certificates worth ~ 20 USD for each time point.

### Measures

#### Experimental Paradigm: The Eye-Preference Task

**Fig. 1:**
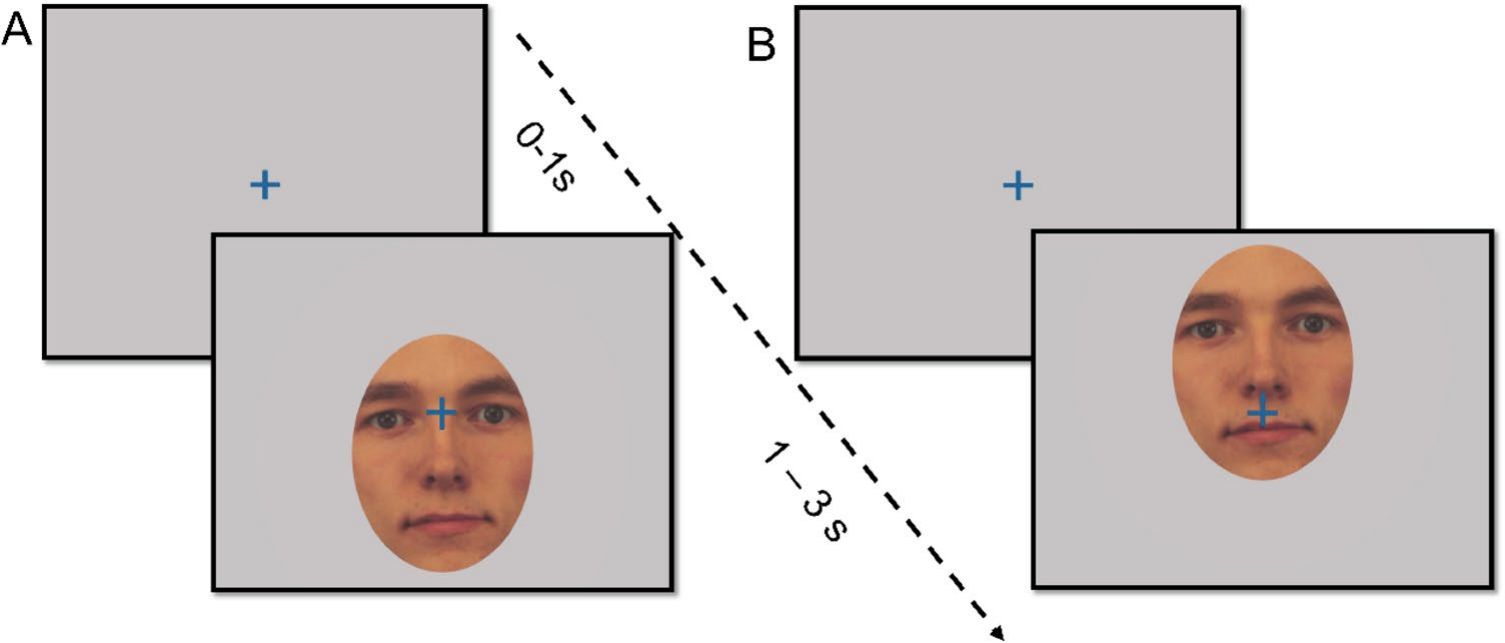
Stimuli and overview of the experiment. All trials started with a fixation cross presented for 1 s followed by the stimulus image presented for 2 s. Stimulus images were positioned relative to the fixation cross so that participants initial point of gaze was either within the eye region (A, Orient-away condition, OA) or in the mouth region (B, Orient-towards condition, OT). Dependent variables were the latency to orient away from eyes (OA) or towards the eyes (OT)

The experimental paradigm was adapted from a previous study [20]. Stimulus images were human faces cropped to show only the inner region of the face. Each trial started with a fixation cross presented for one second before the onset of the stimulus image (see Fig. 1). The stimulus image was then presented, positioned in relation to the fixation cross so that the participant was looking either in the eye region (50% of trials) or in the mouth region (50% of trials). Stimulus images were shown for 2 s. Participants completed 30 trials in each condition (OA and OT), mixed together in a randomized order, interleaved with stimuli from other tasks not analyzed here, which did not include faces. The stimulus images were equally balanced between three emotional expressions: angry, happy, and neutral. The children were instructed to just watch what happened on the screen. The eye-tracking session lasted approximately five minutes.

##### Recording and Analysis of Eye-Tracking Data

Eye-tracking data were recorded at a sample rate of 60 HZ using a corneal-reflection eye tracker (Tobii TX120, Tobii, Danderyd, Sweden). Saccades were identified using a custom I-VT filter with velocity threshold set to 30°/s. Saccadic latency was defined as the time of onset of the first saccade away from the initial cued point of gaze. Two variables were extracted: 1) OA, the latency to orient away from the eyes (and to the mouth) when the initial point of gaze was cued to the eyes, and 2) OT, the latency to initiate/orient a saccade towards the eyes when the initial point of gaze was cued to the mouth. Trials were discarded if participants were not looking at the fixation cross at the onset of the facial image, or if at least 25% valid fixation time was registered at either the primed or the non-primed region. Cut-off was set to 10 valid trials of OA and OT respectively for inclusion in analyzes (discarding five participants on OA and four on OT). Invalid trials could result from movement artefacts, excessive blinks or equipment failure. Setting the cut-off at 8, 10, or 12 did not affect the results significantly.

After exclusion, two complementary eye tracking metrics can be calculated: the latency to initiate a saccade to the non-primed region and the proportion of trials in which a saccade to that region is initiated (as opposed to trials during which gaze is maintained at the primed region). Preliminary analyses in the present sample showed strong overlap between these variables in the OA (*r* = -0.95) and OT (*r* = -0.91) both *p* < 0.0001, in that individuals who were quicker to orient to the non-primed region were also more likely to do so, rather than to maintain their gaze at the primed region. Due to the substantial overlap between these variables, only the latency measures were included in the current study. Cronbach’s alpha was α = 0.93 for OA and α = 0.67 for OT, the former considered excellent and the latter in the lower range for what may be considered acceptable.

#### Symptom Measures at T1 and T2

##### ADHD Symptoms

We used the ADHD Rating Scale-5 for Children and Adolescents [34] to assess ADHD symptoms at T1 and T2. Parents rated the 18 symptoms listed in the DSM-5 [24] on a scale from 0 to 3. Nine items concern inattention and 9 concern hyperactivity/impulsivity. We used the mean of each symptom domain, which were analyzed separately due to its different developmental trajectories [30]. Cronbach’s alpha was α = 0.94 to 0.96.

##### Externalizing Symptoms

We used the Swanson, Nolan, and Pelham scale-IV (SNAP-IV) ODD subscale [35] to assess ODD symptoms. Six age-appropriate DSM-5 symptoms of CD was added to the scale. Parents rated the eight ODD symptoms and six CD symptoms on a scale from 0 to 3. To obtain a broad measure of externalizing behaviors we used the mean across the two domains, which were significantly correlated (*rho* = 0.55, *p* < 0.0001 at T1 and *rho* = 0.38, *p* = 0.001 at T2). Cronbach’s alpha was α = 0.88 at both time points.

##### Generalized Anxiety

We used the generalized-anxiety subscale (comprising 6 items) from Spence Children’s Anxiety Scale (SCAS) [36] to assess anxiety in the children. Parents rated anxiety levels in their children on a scale from 0 to 3. Cronbach’s alpha was α = 0.77 at T1 and α = 0.86 at T2.

##### Change Scores

Change scores were calculated for each symptom domain by subtracting scores at T1 from T2. As such, values > 0 reflect an increase of symptoms across time and values < 0 reflect a symptom decrease.

#### Control Variables

Sex, age, intelligence quotient (IQ) and SES were included as control variables in the models if significantly correlated with any of the main study variables.

##### Intelligence Quotient

We used Block design and Information from WISC-IV [37] as a proxy for IQ. These subtests had the highest loading on non-verbal and verbal IQ in the Swedish validation of WISC-IV [37]. The scaled scores (mean = 10; SD = 3) for the two tests were significantly correlated (*r* = 0.35, *p* = 0.002) and collapsed into one measure for IQ.

##### Socio-Economic Status

Parents rated their own and the child’s other parent’s highest level of education (1 = elementary school, 2 = vocational high school, 3 = theoretical high school, 4 = post high-school studies [not University], and 5 = college/University) and yearly income (1 =  > 10,000 USD; 2 = 10,000 to 20,000 USD; 3 = 20,000 to 30,000 USD, 4 = 30,000 to 40,000 USD, 5 = 40,000 to 50,000, and 6 =  > 50,000 USD). The mean across both parents was used as a proxy for SES.

### Analytic Strategy

The analysis plan was pre-registered in the Open Science Framework (https://osf.io/tg8mw/). Analyzes were performed in IBM SPSS 26 (IBM, Corp., Armonk, NY, USA). *Z* score ± 3 were considered outliers and as such winsorized, that is replaced with the highest/lowest values not considered outliers [38]. Skewness and kurtosis were examined using guidelines by Field [39]. Residuals were plotted and visually inspected for deviant patterns to ensure that linear regressions could be used. Cronbach’s alpha was used to examine internal consistency of the scales and eye tracking variables. Due to a relatively small sample size, the possibility of imputing missing values was scrutinized to increase power. We present descriptive statistics based on original data. All further analyzes are carried out on imputed data. Group differences based on independent samples *t* tests are presented for descriptive purposes. To increase power and based on a dimensional perspective on ADHD, all main analyses are dimensional and carried out on the full sample. We used correlations to examine bivariate associations and hierarchical regression analyses to examine independent contributions. To reduce the number of predictors in the models, only predictors and covariates with significant correlations to a predictor or criterion variable were included in the models. We report unstandardized *b*s as output.

## Results

### Preliminary Results

Ten outliers (representing eight different measures from eight different participants) were found and winsorized. Most measures had excessive skewness, kurtosis, or both. Hence, Spearman correlations were used. Visual inspection of residual plots revealed no deviant patterns and therefore parametric regressions could be used. Nine percent of the data were missing. Little’s MCAR test was non-significant (*p* = 0.071), indicating that data was missing completely at random and five sets of imputations were made using standard fully conditional specification (FCS-Standard) [40], in which all study variables were used in the imputation phase. See Table 1 for descriptive statistics and Table 2 for correlations. OA regarding all emotions were significantly correlated (*rho*s = 0.30 to 0.58, *p*s < 0.05) and collapsed to one measure for OA. OT regarding all emotions were also significantly correlated (*rho* = 0.42 to 0.61, *p*s < 0.05) and collapsed to one measure for OT. As most of these correlations were in the moderate range, unity and diversity of orienting as a function of emotion seem to be present. As such, analyses were conducted with both total scores of OA and OT as well as with scores for the respective emotions. As shown in Table 1, mean symptom levels decreased slightly from T1 to T2 for all symptom domains at the group level. The largest decrease was a reduction of hyperactivity/impulsivity for the ADHD group, which was significantly larger than the reduction for the typically developing children.

**Table 1:** Descriptive statistics

	Total sample			ADHD group			Control group			Group difference
	*n*	*M*	*SD*	*n*	*M*	*SD*	*n*	*M*	*SD*	*p*
SES	81	4.11	1.01	33	3.84	1.02	48	4.29	0.98	.051
IQ	78	9.66	2.57	32	9.52	3.25	46	9.76	2.01	.681
OA Anger	77	768	287	33	807	311	44	705	264	.106
OA Happy	81	762	324	34	789	311	47	744	336	.537
OA Neutral	71	804	346	26	884	354	45	762	337	.170
OA	81	769	241	34	803	236	47	746	244	.279
OT Anger	81	436	223	34	476	267	47	407	183	.197
OT Happy	79	491	244	31	525	277	48	453	218	.095
OT Neutral	82	394	181	34	395	196	48	393	171	.967
OT	82	442	187	34	476	209	48	417	168	.159
T1 Inatt	81	0.99	0.81	33	1.78	0.60	48	0.44	0.33	** < .001**
T1 Hyp/Imp	81	0.86	0.83	33	1.67	0.67	48	0.29	0.28	** < .001**
T1 ODD/CD	81	0.44	0.41	33	0.72	0.47	48	0.26	0.22	** < .001**
T1 GAD	79	1.66	0.42	33	1.79	0.47	46	1.57	0.36	**.020**
T2 Inatt	67	0.94	0.75	27	1.58	0.65	40	0.45	0.37	** < .001**
T2 Hyp/Imp	67	0.67	0.72	27	1.25	0.68	40	0.28	0.41	** < .001**
T2 ODD/CD	67	0.38	0.37	27	0.55	0.44	40	0.23	0.25	**.011**
T2 GAD	67	1.62	0.52	27	1.80	0.61	40	1.52	0.41	**.031**
Inatt CS	67	–0.04	0.43	27	-0.15	0.55	40	0.04	0.31	.112
Hyp/Imp CS	67	–0.17	0.43	27	-0.45	0.50	40	-0.02	0.28	** < .001**
ODD/CD CS	67	–0.06	0.26	27	-0.11	0.33	40	-0.02	0.20	.197
GAD CS	65	–0.05	0.35	27	0.03	0.37	38	-0.08	0.34	.235

*SES* socio-economic status, *OA* orienting away from eyes, *OT* orienting towards eyes, *Inatt* inattention, *Hyp/Imp* hyperactivity/impulsivity, *ODD/CD* oppositional defiant disorder/conduct disorder, *CS* change score (across T1 and T2). OA and OT are measured in milliseconds; all other measures are based on mean values from the rating scales

**Table 2:** Bivariate correlations between all study variables

	1	2	3	4	5	6	7	8	9	10	11	12	13	14	15	16	17	18	19	20	21	22	23	24
1. SES	1	.30^**^	.15	-.03	-.06	.05	.22^†^	.04	-.13	-.09	-.12	-.13	-.25*	-.29*	-.27*	-.27*	-.27*	-.34**	-.18	-.24*	-.00	-.03	-.01	-.05
2. IQ		1	.15	-.13	-.09	-.12	.00	-.13	-.05	-.04	-.15	-.11	-.18	-.12	-.08	-.30**	-.23^†^	-.26*	-.19	-.26*	.03	-.19	-.11	-.00
3. Age			1	-.06	.26^*^	.22^†^	.18	.32^**^	-.04	-.20^†^	-.16	-.16	.09	-.04	-.17	-.07	.06	-.16	-.05	-.05	.00	-.13	.19	.02
4. Sex				1	-.01	.00	-.11	-.05	.03	.08	.17	.16	-.06	.06	.17	.14	-.00	.06	.17	.25^*^	.11	-.04	-.05	.18
5. OA anger					1	.57^***^	.33^**^	.79^***^	-.10	-.17	-.21^†^	-.18	.34^**^	.30^*^	.15	.07	.38^**^	.23	.21^†^	.10	-.09	-.17	.10	.15
6. OA happy						1	.32^**^	.86^***^	-.08	-.14	-.16	-.15	.26^*^	.11	.14	-.02	.31^*^	.13	.28^*^	-.04	.02	-.01	.16	.03
7. OA neutral							1	.58^***^	-.14	-.18	-.21^†^	-.16	.06	-.01	.06	-.15	.08	-.07	.13	-.27^*^	.05	-.07	.00	-.13
8. OA								1	-.15	-.24*	-.27^*^	-.25^*^	.28^*^	.15	.17	-.06	.35^**^	.12	.30^*^	-.07	.02	-.08	.14	.06
9. OT anger									1	.60^***^	.47^***^	.79^***^	.20^†^	.11	.04	-.05	.05	.06	.09	-.03	-.18	-.15	-.00	-.04
10. OT happy										1	.60^***^	.87^***^	.19	.19	-.03	.04	.06	.07	-.06	.07	-.14	-.15	-.07	.04
11. OT neutral											1	.81^***^	.09	.13	-.11	-.02	.02	.11	-.03	.02	-.12	-.06	.04	.06
12. OT												1	.21^†^	.20^†^	.01	.02	.04	.10	.05	.03	-.21	-.16	-.04	.01
13. T1 Inatt													1	.79^***^	.52^***^	.20^†^	.75^***^	.60^***^	.46^***^	.25^*^	-.38^**^	-.30^**^	-.10	.17
14. T1 Hyp/Imp														1	.59^***^	.26^*^	.68^***^	.75^***^	.50^***^	.29^*^	-.18	-.43^***^	-.17	.21^†^
15. T1 ODD/CD															1	.27^*^	.44^***^	.52^***^	.70^***^	.26^*^	-.09	-.15	-.38^**^	.13
16. T1 GAD																1	.16	.24^*^	.24^*^	.64^***^	-.13	-.09	-.11	-.15
17. T2 Inatt																	1	.77^***^	.53^***^	.23^†^	.15	-.17	.10	.20
18. T2 Hyp/Imp																		1	.58^***^	.34^*^	-.01	-.01	.07	.27^†^
19. T2 ODD/CD																			1	.23^†^	-.01	-.06	.31^*^	.11
20. T2 GAD																				1	-.07	.00	-.06	.60^***^
21. Inatt CS																					1	.48^***^	.09	.06
22. yp/Imp CS																						1	.16	.07
23. ODD/CD CS																							1	.02
24.GAD CS																								1

*OA* orienting away from eyes, *OT* orienting towards eyes, *Inatt* inattention, *Hyp/Imp* hyperactivity/impulsivity, *ODD/CD* oppositional defiant disorder/conduct disorder, *CS* change score (across T1 and T2)

^†^*p* < .10; *p < .05; **p < .01; ***p < .001

At group level, participants oriented slower from the eyes (OA) of neutral than happy (t(76) = 6.82, *p* < 0.001) and angry faces (t(76) = 4.40, *p* < 0.001), whereas no difference was found between happy and angry faces (t(76) = 0.95, *p* = 0.340). When primed to look at the mouth (OT), participants were slower to orient to the eyes of happy than angry (t(76) = 3.24, *p* = 0.002) and neutral (t(76) = 2.92, *p* = 0.005) faces. No difference was found between angry and neutral faces (t(76) = 0.34, *p* = 0.736).

### Concurrent Associations Between Eye Preference and Symptoms

OA was significantly correlated with inattention at T1 (*rho* = 0.28, *p* = 0.018), in that a longer latency to orient away from the eyes (when primed to look at the eyes) was associated with more symptoms. This was the case for OA anger (*rho* = 0.34, *p* = 0.003) and OA happy (*rho* = 0.26, *p* = 0.024). Further, OA anger was significantly correlated with hyperactivity/impulsivity (*rho* = 0.30, *p* = 0.010). OT was not significantly related to concurrent symptom levels.

To examine specific relations between eye preference and symptoms, we performed a hierarchical regression model with OA as criterion variable and age, SES, hyperactivity/impulsivity and ODD/CD as covariates entered in the first step and inattention as the main predictor entered in the final step. This was informed by the pattern of significant correlations, where inattention was the only predictor with a significant correlation with OA. The results show that inattention was independently associated with OA (see Table 3). As OA anger and OA happy were both significantly correlated with inattention, we ran an exploratory analysis to examine independent effects of emotion in that we regressed inattention on OA anger and OA happy. The results showed that OA anger (*b* = 0.001, *p* = 0.018, R^2^Δ = 0.083) was independently associated with inattention at T1 whereas happy was not (*p* = 0.693).

**Table 3:** The final steps of hierarchical regression models with latency to orient away from eyes (OA) as criterion variable

	Predictors at T1				Predictors at T2			
	*b*	*p*	Adjusted R^2^	R^2^Δ	*b*	*p*	Adjusted R^2^	R^2^Δ
Step 2			0.154				0.098	
Constant	105.39	.645			171.97	.543		
Age	37.71	.059			45.09	.037		
SES	42.74	.107			46.69	.109		
Hyp/Imp	− 77.05	.228			− 88.13	.277		
GAD					− 26.86	.649		
ODD/CD	83.34	.327			79.10	.372		
Inattention	**136.04**	**.037**		.050	**129.32**	**.049**		.049

*SES* socio-economic status, *Hyp/Imp* hyperactivity/impulsivity, *GAD* symptoms of generalized anxiety disorder, *ODD/CD* symptoms of oppositional defiant disorder/conduct disorder. Significant results in bold

### Longitudinal Associations Between Eye Preference at T1 and Symptoms at T2

OA was significantly correlated with inattention (*rho* = 0.35, *p* = 0.005) and ODD/CD (*rho* = 0.30, *p* = 0.017) at T2, in that a longer latency to orient away from the eyes was associated with more symptoms two years later. As for specific emotions, inattention at T2 was significantly correlated with OA anger (*rho* = 0.38, *p* = 0.005) and OA happy (*rho* = 0.31, *p* = 0.016) and OA happy was significantly correlated with ODD/CD at T2 (*rho* = 0.28, *p* = 0.018). Further, OA neutral was significantly correlated with generalized anxiety (*rho* = -0.27, *p* = 0.045), in that a shorter latency to look away from neutral faces was associated with more symptoms. OT was not significantly related to symptom levels at T2.

To examine specific relations between eye preference and symptoms at T2, we performed a hierarchical regression model with OA as criterion variable, and age, SES, hyperactivity/impulsivity, and GAD as covariates entered in the first step and inattention and ODD/CD as the main predictors entered in the final step. The results show that inattention was independently associated with OA whereas ODD/CD was not (see Table 3). Exploratory analyses regarding specific emotions showed that when included in the same step in a hierarchical regression, neither OA anger nor OA happy made independent contributions (*p*s < 0.130) to inattention at T2, indicating a common rather than specific effects of emotion.

OA and OT were unrelated to change in symptoms across T1 and T2 (see Table 2). As attention to eyes was unrelated to change in symptoms we did not perform additional analyzes. We conclude that this finding indicates that altered attention to eyes does not exacerbate symptoms over time.

## Discussion

In the current study, we set out to examine concurrent and longitudinal relations between attention to other’s eyes and core symptoms of ADHD and comorbid externalizing (ODD/CD) and internalizing (generalized anxiety) symptoms. Our main results indicate that a longer latency to orient away from the eyes, when primed to look at the eye region was associated with higher levels of inattentive symptoms. This was found both concurrently and at a two-year follow up, and remained with control for the other symptom domains. This association was present for emotional (angry and happy) but not neutral faces and seems primarily driven by a tendency to ‘get stuck’ in the eye region of angry faces, which was also related to concurrent high levels of hyperactivity/impulsivity. The latency to orient towards the eyes when primed to look at the mouth was unrelated to symptom levels, indicating that our finding is not a sign of a general reduced orienting speed. Attention to the eyes was not associated with exacerbation of symptoms across time.

Together, these results demonstrate that ADHD symptoms in childhood are linked to alterations in the temporal microstructure of social attention. Adaptive face processing requires a quick and coordinated sequence of gaze shifts between facial regions and objects in the environment, and disruptions to any stage of this process could potentially lead to social interaction impairments. Interestingly, whereas the latency to orient *away* from eyes was specifically linked to symptoms of inattention both concurrently and longitudinally, no links were found to the latency to *seek* eye contact. These two stages in the sequence of social attention have different neurodevelopmental correlates. Rapid orienting to eyes is observed already in infancy and is believed to be driven by a largely subcortical network of brain regions sensitive to the coarse visual characteristics of eyes [14, 41, 42]. Disruptions at this stage have been linked to autism [43–45] (but see [46]) and Williams syndrome, a genetic disorder with early emerging social alterations [47]. In contrast, the latency to reorient from eyes is believed to be modulated by higher order cognitive processes including spatial attention and motivation. Delayed reorienting from eyes in children with higher levels of ADHD symptoms therefore seemingly indicates disruption to this normative process. A similar pattern of attention was recently reported in a study of social anxiety disorder (SAD) in children aged 10–17 years using the same paradigm as the current study [20]. Here, children with SAD were slower than healthy controls to orient from the eye region during the earliest time stages of attention but did not differ in the latency to orient to eyes. As such, different alterations of face processing may be present for ADHD and ASD, whereas ADHD and SAD may overlap on a phenotypical level. Importantly, symptoms of SAD and inattention are overlapping, and may share similar aetiological factors [48]. It should be noted that, at the group level, children in the present study were quicker to reorient from the eyes when viewing emotional than neutral faces.

A longer latency to orient away from the eyes was consistently and independently associated with inattentive symptoms, concurrently and longitudinally, which indicates a robust relation. Further, this association was most pronounced for angry faces. These results are aligned with attenuated tonic arousal as a potential mechanism underlying our result. Several previous studies have reported both behavioral and physiological evidence for hypoarousal [33, 49–51], and hypoarousal has been suggested to underlie enhanced attention to other’s eyes in other developmental disorders [52]. A second potential mechanism explaining this result, is impaired disengagement of attention from stimuli eliciting emotional arousal. In support of this interpretation, previous studies reported similar effects in youth with rumination, a population which like ADHD is associated with reduced cognitive control [53], and recently also in social anxiety disorder [20]. Of note, delayed orienting away from angry faces was also concurrently (but not longitudinally) related to hyperactivity/impulsivity. The lack of a prospective relation to hyperactivity/impulsivity may be explained by a commonly observed developmental decline in this symptom dimension [30].

In contrast to our hypotheses, we did not find a specific connection between attention to eyes and comorbid externalizing or internalizing symptoms. As our sample was oversampled for ADHD diagnoses rather than high levels of comorbid symptoms, we may not have had sufficient power to detect such an effect due to a restricted range and variation for these measures. There were however prospective associations between longer orienting away from eyes and symptoms of ODD/CD at T2, suggesting a link between the two. This link seems to be shared or overlapping with ADHD symptoms at T2, proposing a general rather than specific association between these constructs. In sum, our results propose a specific association to inattention rather than to comorbid symptoms, which may be secondary to symptoms of ADHD, but not to attention to other’s eyes.

The lack of an exacerbating effect suggests that deviant eye gaze does not contribute to escalation of symptoms over time. This may infer that eye gaze on this time scale is not involved in pro-social learning (hence no connection to change in ODD/CD) or emotion processing (hence no connection to generalized anxiety). Rather, our results may suggest that altered eye gaze in ADHD could be a trait-like factor that persists over time but does not affect symptom levels over time.

An interesting question for future studies is whether altered eye gaze processing in ADHD can be modulated by stimulant- and non-stimulant medication and other treatments. Previous studies have shown that both types of treatment can reduce core symptoms of ADHD, as well as associated comorbidity [54]. However, individual variability exists and effects on social skills and peer interaction difficulties are not sufficiently studied. It is not known whether proximal changes in social attention can mediate more distant treatment effects. If such relations would be found, social attention metrics could be feasible as biomarkers in treatment research. It should be noted that, in addition to its links to ADHD symptoms, the OA measure in the present study showed excellent internal psychometric consistency (Cronbach’s α = 0.93), suggesting that it may be feasible as a marker task in treatment research. The OT measure, however, had lower internal consistency (Cronbach’s α = 0.67), which calls for further examination before it can be proposed as a reliable measure in future studies. The role of social attention metrics in diagnostic assessments should be scrutinized, as subtyping individuals with or without these impairments could guide tailored treatments. Futures studies should also directly compare children with ADHD and autism, since previous studies have linked the latter condition to atypical eye contact [43].

As for limitations, we wish to acknowledge that symptom ratings from multiple informants (such as teachers, parents and the affected children themselves) could have given a more complete picture than parent-ratings alone. Relatedly, self-ratings of internalizing could potentially have provided better sensitivity. A broader assessment of internalizing symptoms would have been informative, as that would have enabled a distinction between social and non-social aspects of anxiety. In addition, the relatively low variation in comorbid symptoms is a limitation and we do not have information about which interventions (medical, psychological or pedagogical) our subjects have received between T1 and T2. Earlier and repeated assessment of eye-preference could further inform on the effect of gaze on pro-social learning and emotion processing. Finally, we did not correct for multiple testing, based on the increased risk of making type-2 errors with such a modest sample. Instead, we believe that our pre-registration of analyses reduces the risk of providing false-positive results.

### Summary

The current study explored the foundation of impaired social interactions in ADHD. We examined associations between attention to other’s eyes and symptoms of core ADHD symptoms and comorbid externalizing and internalizing symptoms in a sample of 82 eight to 13-year-olds oversampled for ADHD. The latency to a first gaze shift to and away from the eye region, when primed to looked at the eyes or the mouth, was recorded with eye tracking. The main results show that longer looking at the eye region before reorienting was independently associated with concurrent and longitudinal symptoms of inattention with control for the other symptom domains, and significantly correlated with inattention and externalizing symptoms two years later. The results propose that the temporal microstructure of attention to other’s eyes is altered in children with symptoms of ADHD, which in turn may contribute to social impairments.
